# Molecular and comparative analysis of *Salmonella enterica *Senftenberg from humans and animals using PFGE, MLST and NARMS

**DOI:** 10.1186/1471-2180-11-153

**Published:** 2011-06-27

**Authors:** Ryan M Stepan, Julie S Sherwood, Shana R Petermann, Catherine M Logue

**Affiliations:** 1 Department of Veterinary and Microbiological Sciences, North Dakota State University, Fargo, ND 58108, USA; 2 Department of Veterinary Microbiology and Preventive Medicine, College of Veterinary Medicine, 1802 University Blvd VMRI #5, Iowa State University, Ames IA, 50011-1240, USA

## Abstract

**Background:**

*Salmonella *species are recognized worldwide as a significant cause of human and animal disease. In this study the molecular profiles and characteristics of *Salmonella enterica *Senftenberg isolated from human cases of illness and those recovered from healthy or diagnostic cases in animals were assessed. Included in the study was a comparison with our own sequenced strain of *S. *Senfteberg recovered from production turkeys in North Dakota. Isolates examined in this study were subjected to antimicrobial susceptibility profiling using the National Antimicrobial Resistance Monitoring System (NARMS) panel which tested susceptibility to 15 different antimicrobial agents. The molecular profiles of all isolates were determined using Pulsed Field Gel Electrophoresis (PFGE) and the sequence types of the strains were obtained using Multi-Locus Sequence Type (MLST) analysis based on amplification and sequence interrogation of seven housekeeping genes (*aroC*, *dnaN*, *hemD*, *hisD*, *purE*, *sucA*, and *thrA*). PFGE data was input into BioNumerics analysis software to generate a dendrogram of relatedness among the strains.

**Results:**

The study found 93 profiles among 98 *S*. Senftenberg isolates tested and there were primarily two sequence types associated with humans and animals (ST185 and ST14) with overlap observed in all host types suggesting that the distribution of *S. *Senftenberg sequence types is not host dependent. Antimicrobial resistance was observed among the animal strains, however no resistance was detected in human isolates suggesting that animal husbandry has a significant influence on the selection and promotion of antimicrobial resistance.

**Conclusion:**

The data demonstrates the circulation of at least two strain types in both animal and human health suggesting that *S. *Senftenberg is relatively homogeneous in its distribution. The data generated in this study could be used towards defining a pathotype for this serovar.

## Introduction

*Salmonella *species are recognized as agents of illness and disease in both humans and animals with greater than 2000 serotypes recognized; the gastrointestinal tract of animals is considered the primary reservoir of the pathogen with human illness usually linked to exposure to contaminated animal-derived products such as meat or poultry [1,2]. Annually in the US *Salmonella *is estimated to cause approximately 1 million illnesses, 19,000 hospitalizations and approximately 378 deaths [3]. While some of these *Salmonella *species are commonly implicated in human and animal disease there are emerging strains that are also gaining recognition. The annual list from the CDC now includes exotic strain types not previously recognized. From 2007 data, the CDC estimates that *Salmonella *species account for approximately 20% of suspected outbreaks and greater than 3500 illnesses among the sentinel states (http://www.cdc.gov/mmwr/preview/mmwrhtml/mm5931a1.htm?s_cid=mm5931a1_w). Although *S. *Senftenberg is not listed among the top 20 serotypes implicated in human illness [4] the organism is routinely detected in humans and has been recognized in clinical non-human cases of disease (ranked #10 in 2006) and in non-clinical non-human cases (ranked #4), supporting the potential for the emergence of this strain type in human disease.

An important aspect in the characterization of pathogens is an assessment of the role of molecular analysis in determining clonal and strain distribution across various environments and hosts. While there are a range of methods available for strain characterization and sub-typing, the most commonly used methods include Pulse Field Gel Electrophoresis (PFGE) [5-8], Multi-Locus Sequence Type (MLST) analysis [6,9,10], and virulence or resistance gene carriage [11-13]. In addition, phenotypical analysis includes trait expression through antimicrobial susceptibility analysis or phenotype microarray type analysis [1,14,15].

PFGE has become a powerful tool in assessing the genetic relatedness of strains and is commonly used by the CDC, USDA and other federal agencies for assessing strains implicated in both human and animal disease and outbreaks associated with a particular pathogen. The method involves selective restriction of the genome and analysis of fragment patterns using a pulsed electric field. Restriction patterns generated are compared to controls strains and each other using cluster analysis software [6,16]. While PFGE offers great power in comparative analysis and is relatively useful for visual representation of strain differences, it can suffer limitations. Not all strains may restrict well or will not restrict with specified enzymes and the time required for preparation and analysis can be intensive [17,18]. Others have reported that PFGE may have limited discriminatory power in subtyping certain highly clonal serotypes such as *S*. Enteriditis and *S*. Hadar [19] and may require multiple enzymes to be of benefit [20].

Multi-Locus Sequence Type (MLST) analysis is also useful as a tool in molecular analysis - it uses the approach of allelic differences in the sequence of various house-keeping genes which can be exploited to differentiate strains [6,21,22]. Although the method is relatively straightforward, (involving amplification of housekeeping genes by PCR followed by sequencing and interrogation of the sequences against a database to determine sequence types) there are however, limitations in its ability to differentiate strains and may not be useful where all strains being tested are of the same serotype. Fakhr et al [5] found that PFGE provided greater strain differentiation among *S. *Typhimurium isolates compared to MLST analysis for the genes *manB*, *pduF*, *glnA*, and *spaM *and found no nucleotide differences among 85 strains tested from cattle. The study suggested that genes of greater variation were necessary to ensure the power of MLST as a differentiation tool such as those of virulence [5,23]. In a recent study Liu et al [24] noted that an MLST analysis based on the two genes *sseL *and *fimH *for *S. enterica *species was congruent with serotypes. An alternative approach to MLST housekeeping genes has been the use of an MLST associated with virulence genes such as MVLST [5,6,23] which has proven successful for *Listeria spp *[25,26], but currently does not appear to be as well established for *Salmonella *spp or other gram negative organisms.

Molecular profiling of *Salmonella *has been carried out by a number of authors in an attempt to determine strain types and their distribution in human or animal hosts and relatedness [7,27-32]. Such approaches have been useful in assessing the role of specific serotypes in human and animal disease and assessing overlap between the hosts. In this study, the molecular profiles and characteristics of *Salmonella enterica *Senftenberg from humans and animals were assessed to determine the distribution of the strain type across the different host species and to assess the relatedness of *S*. Senftenberg strains circulating in animals and humans.

## Materials and methods

### Isolates studied

All animal isolates of *S. enterica *Senftenberg used in this study were obtained from the lab collection of Logue, the North Dakota Veterinary Diagnostic Lab (ND VDL, Fargo, ND), and the National Veterinary Services Laboratory (NVSL, Ames, IA) and represented strains from ND and various states in the US. Human isolates *S. *Senftenberg were obtained from the Centers for Disease Control (CDC, Atlanta, GA) and represented a collection of isolates from human cases of salmonellosis across the United States. All isolates were stored frozen at -80°C in Brain Heart Infusion (BHI, Difco, Sparks, MD) broth supplemented with 20% glycerol. Passaging of the strains was kept to a minimum in order to preserve isolate integrity. In total, 71 isolates from animals, 22 from humans and 5 isolates from feed and goose down were used in this study.

### NARMS analysis

All isolates were subjected to antimicrobial susceptibility testing using the broth microdilution method and the National Antimicrobial Resistance Monitoring Scheme (NARMS) panels (CMV1AGNF, Sensititre^®^, Trek Diagnostics, Cleveland, OH), according to the Clinical Laboratory Standards Institute [33] guidelines. The panel tested antimicrobial susceptibility to the following antimicrobials: amikacin (0.5 - 64 μg/ml), ampicillin (1 - 32 μg/ml), amoxicillin/clavulanic acid (1/0.5 - 32/16 μg/ml), ceftriaxone (0.25 - 64 μg/ml), chloramphenicol (2 - 32 μg/ml), ciprofloxacin (0.015 - 4 μg/ml), trimethoprim/sulfamethoxazole (0.12/2.38 - 4/76 μg/ml), cefoxitin (0.5 - 32 μg/ml), gentamicin (0.25 - 16 μg/ml), kanamycin (8 - 64 μg/ml), nalidixic acid (0.5 - 32 μg/ml), sulfisoxazole (15-256 μg/ml), streptomycin (32 - 64 μg/ml), tetracycline (4 - 32 μg/ml), and ceftiofur (0.12 - 8 μg/ml).

*Salmonella *isolates were recovered from frozen stock to Tryptone Soy Agar (TSA) and incubated at 37°C for 18-24 h; cell suspensions were prepared and adjusted to a 0.5 McFarland standard. Then, 10 μl of the suspension was added to 11 ml of Mueller-Hinton broth (Trek Diagnostics) and mixed; the NARMS panels were inoculated using the Sensititre^® ^Autoinoculator (Trek Diagnostics) following the manufacturer's instructions. The plates were sealed and incubated at 37°C for 18 h. After incubation, the plates were read using the Sensititre Autoreader (Trek Diagnostics) to record growth or no growth of the isolates in each of the wells. The minimum inhibitory concentration (MIC) was recorded for each isolate and compared to breakpoints that were defined by the CLSI. A breakpoint is defined as the minimum concentration of antimicrobial above which growth should not occur [34]. Breakpoints used in this study are indicated in the results section. CLSI specified positive control strain *Escherichia coli *ATCC 25922 was used to ensure the efficacy of the procedure for *Salmonella*. The isolates were recorded as resistant or sensitive for each antimicrobial according to breakpoints specified by CLSI [33].

### PFGE analysis

Pulsed Field Gel Electrophoresis (PFGE) was performed as previously described [35] with slight modifications. *Salmonella enterica *serotype Braenderup H9812 (ATCC #BAA-664) was used as the molecular weight size standard. Restriction endonuclease digestion was carried out using 25 U *Xba*I (Invitrogen, Carlsbad, CA) in a final volume of 100 μl at 37°C for 3 h. DNA macrorestriction fragments were resolved over 18 h on 1% SeaKem Gold Agarose (Cambrex, Rockland, ME) (in 0.5X TBE) using the Chef Mapper XA system (Bio-Rad, Hercules, CA) auto algorithm function for a low molecular weight of 30 kb and a high molecular weight of 600 kb. Gels were stained in 1 μg ethidium bromide ml^-1 ^in reagent grade water for 30 min, with washes as needed and the restriction patterns visualized by UV transillumination using an Alpha Innotech Imager (Alpha Innotech, Santa Clara, CA).

Macrorestriction patterns were compared using the BioNumerics Fingerprinting software (Version 6.5, Applied Math, Austin, TX). The similarity index of the isolates was calculated using the Dice correlation coefficient option of the software with a position tolerance of 1% and an optimization of 0.5%. The unweighted-pair group method using average linkages (UPGMA) was used to construct a dendrogram. Generation of the dendrogram was based on a single experiment analysis (PFGE only) and was not weighted to include sequence type information from the MLST analysis or the antimicrobial resistance phenotype data.

### MLST analysis

Multi locus sequence type analysis was carried out using the MLST protocols described at the MLST website (http://mlst.ucc.ie/mlst/dbs/Senterica/documents/primersEnterica_html). Briefly, all isolates were struck to TSA and incubated at 37°C for 18-24 h. Following incubation, colonies were picked to 40 μl of single cell lysing buffer (50 μg/ml of Proteinase K (Amresco, Solon, OH) in TE buffer (pH8)), the cells were lysed by heating to 80°C for 10 minutes followed by 55°C for 10 minutes in a thermocycler (Eppendorf, Hamburg, Germany). The final suspension was diluted 1:2 in sterile water, centrifuged to remove cellular debris and transferred to a sterile tube [36]. DNA from each test strain was stored frozen at -18°C until use.

PCR amplification for the genes of the MLST panel was carried out as follows. Primer pairs (table 1) (IDT, Coralville, IA) were used to amplify the DNA for the presence of the following genes *thrA*, *purE*, *sucA*, *hisD*, *aroC*, *hemD *and *dnaN*. All PCR reactions were carried out in 50 μl volumes containing 1 μl of DNA template, *Taq *DNA polymerase (Promega, Madison, WI) (1.25 U), 1 X PCR buffer (Promega), Forward and Reverse primers (0.1 μM) (IDT), and DNTPs (200 μM) (Promega). PCR reactions were carried out in a thermocycler (Eppendorf) using the following cycling parameters 94°C for 30 s; followed by 30 cycles of 95°C for 30 s; 55°C 30 s and 76°C for 30 s, with a final extension of 75°C for 2 min followed by hold at 4°C. 10 μl of the PCR products were loaded into 1% agarose gels in 1 X TAE with EZ Vision One (Amresco) loading dye, and run at 100 v in 1X TAE for 60 minutes. Images of the gels were captured using an Alpha Innotech imager and recorded.

**Table 1:** MLST Primers and PCR Primers used in the amplification of the genes and the expected product sizes used in this study.

*Gene PCR Primers*	*Product size*	*Gene Sequencing Primers*
*thrA*: F 5'-GTCACGGTGATCGATCCGGT-3'	852 bp	*thrA*: sF 5'-ATCCCGGCCGATCACATGAT-3'
*thrA*: R 5'-CACGATATTGATATTAGCCCG-3'		*thrA*: sR 5'-CTCCAGCAGCCCCTCTTTCAG-3'
*thrA*: R1 5'-GTGCGCATACCGTCGCCGAC-3' (also Seq)		
*purE*: F 5'-ATGTCTTCCCGCAATAATCC-3'	510 bp	*purE*: sF 5'-CGCATTATTCCGGCGCGTGT-3'
*purE*: R 5'-TCATAGCGTCCCCCGCGGATC-3'		*purE*: sF1 5'-CGCAATAATCCGGCGCGTGT-3'
*purE*: R1 5'-CGAGAACGCAAACTTGCTTC-3'		*purE*: sR 5'-CGCGGATCGGGATTTTCCAG-3'
		*purE*: sR1 5'-GAACGCAAACTTGCTTCAT-3'
*sucA*: F 5'-AGCACCGAAGAGAAACGCTG-3'	643 bp	*sucA*: sF 5'-AGCACCGAAGAGAAACGCTG-3'
*sucA*: R 5'-GGTTGTTGATAACGATACGTAC-3'		*sucA*: sR 5'-GGTTGTTGATAACGATACGTAC-3'
*hisD*: F 5'-GAAACGTTCCATTCCGCGCAGAC-3'	894 bp	*hisD*: sF 5'-GTCGGTCTGTATATTCCCGG-3'
*hisD*: R 5'-CTGAACGGTCATCCGTTTCTG-3'		*hisD*: sR 5'-GGTAATCGCATCCACCAAATC-3'
*aroC*: F 5'-CCTGGCACCTCGCGCTATAC-3'	826 bp	*aroC*: sF 5'-GGCACCAGTATTGGCCTGCT-3'
*aroC*: R 5'-CCACACACGGATCGTGGCG-3'		*aroC*: sR 5'-CATATGCGCCACAATGTGTTG-3'
*hemD*: F 5'-ATGAGTATTCTGATCACCCG-3'	666 bp	*hemD*: sF 5'-GTGGCCTGGAGTTTTCCACT-3'
*hemD*: F1 5'-GAAGCGTTAGTGAGCCGTCTGCG-3'		*hemD*: sF1 5'-ATTCTGATCACCCGCCCCTC-3'
*hemD*: R 5'-ATCAGCGACCTTAATATCTTGCCA-3'		*hemD*: sR 5'-GACCAATAGCCGACAGCGTAG-3'
*dnaN*: F 5'-ATGAAATTTACCGTTGAACGTGA-3'	833 bp	*dnaN*: sF 5'-CCGATTCTCGGTAACCTGCT-3'
*dnaN*: R 5'-AATTTCTCATTCGAGAGGATTGC-3'		*dnaN*: sR 5'-CCATCCACCAGCTTCGAGGT-3'
*dnaN*: R1 5'-CCGCGGAATTTCTCATTCGAG-3' (also Seq)		

### Sequencing of PCR products

All PCR products obtained above were cleaned and submitted for sequencing as follows. The PCR product was cleaned of amplification primer using the QIAquick^® ^PCR Purification Kit (Qiagen, Valencia, CA) as per manufacturer's instructions. Purified DNA was sequenced at Iowa State University's DNA Facility (Ames, IA) with the sequencing primers for each gene as outlined in table 1. Sequencing was carried out on an Applied Biosystems 3730xl DNA Analyzer (Applied Biosystems, Foster City, CA, USA). Sequence data obtained was imported into DNAStar (Lasergene, Madison, WI), trimmed and aligned to the control sequences (obtained from the MLST site) and interrogated against the MLST database. Sequence types generated were recorded and added to the strain information (see above).

### Strain analysis by Simpson's Index of Diversity

The discriminatory ability of PFGE, antimicrobial resistance profiling, and MLST analysis was calculated using the numerical index of discrimination (*D*) according to the method of Hunter and Gaston [37]. The discriminatory index represents the probability that two unrelated strains sampled from the test population will be placed into different typing groups [37].

## Results

**Figure 1:**
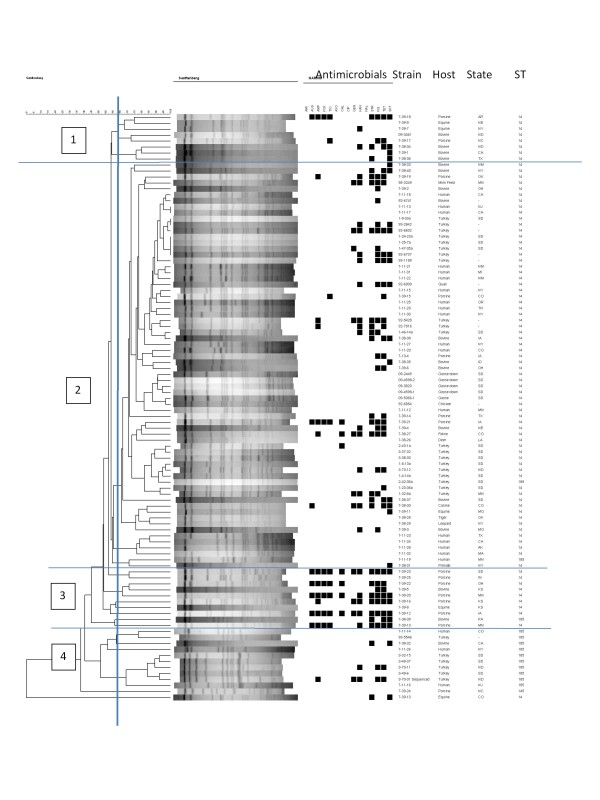
**Dendrogram displaying PFGE profiles, antimicrobial resistance profiles and sequence types (ST) of *S*. Senftenberg from animal and human hosts**. Key for antimicrobial abbreviations - see table 2.

Figure 1 shows the dendrogram analysis of all isolates (n = 98) examined in the study including PFGE profiles, MLST sequence types and antimicrobial susceptibility data of *S. *Senftenberg from human and animal hosts examined in this study. Dendrogram generation was based on PFGE analysis and not weighted for ST or antimicrobial resistance data which are included in the figure.

PFGE analysis identified 93 profiles among the 98 isolates examined. Cluster analysis primarily divided the isolates into four main clusters at approximately 58% similarity. The upper cluster (cluster 1) consisted primarily of porcine, bovine and equine isolates; these were subtyped as ST 14. Cluster 2, the largest cluster, consisted of animal and human isolates and all but one were ST 14. Cluster 3 contained primarily porcine isolates of ST 14; isolates in this cluster also had the highest rates of antimicrobial resistance with most displaying resistance to approximately 10 antimicrobials. Cluster 4 was composed of human and animal isolates (including the sequenced strain) and were all identified as ST 185.

Antimicrobial susceptibility analysis (Table 2) found that all of the human isolates tested were susceptible to all 15 antimicrobial agents. In contrast, the animal strains showed resistance to a range of antimicrobials with the most common resistances observed being to tetracycline, sulfisoxazole, kanamycin, and streptomycin (29.5 to 52.1%). Lower rates of resistance were observed to agents such as amoxicillin/clavulanic acid, ampicillin, cefoxitin, ceftiofur, ceftriaxone, chloramphenicol, gentamicin, and trimethoprim/sulfamethoxazole (range 9.8% to 19.7%). Thirty-three different resistance profiles were observed among the animal isolates (Table 3) with most patterns being represented by one isolate. When examined by host species, the highest rates of resistance were observed for isolates that originated from porcine hosts. Of interest, 13 isolates of porcine origin, 11 bovine and 12 turkey were resistant to two or more antimicrobials. Ten isolates were resistant to one antimicrobial agent and 26 animal isolates (including miscellaneous) were susceptible to all agents tested. Multidrug resistance was also found in one isolate of the following origin: feline, canine, mink feed, quail, and equine.

**Table 2:** Antimicrobial resistance among animal, human and miscellaneous sources of *S*. Senftenberg

Antimicrobial	Breakpoint	Animal (n = 71)	Human (n = 22)	Other (n = 5)
Amikacin (AMI)	≥64	0	0	0
Amoxicillin/Clavulanic Acid (AUG)	≥32/16	7 (9.8%)	0	0
Ampicillin (AMP)	≥32	14 (19.7%)	0	0
Cefoxitin (FOX)	≥32	8 (11.2%)	0	0
Ceftiofur (TIO)	≥8	8 (11.2%)	0	0
Ceftriaxone (AXO)	≥4	8 (11.2%)	0	0
Chloramphenicol (CHL)	≥32	11 (15.4%)	0	0
Ciprofloxacin (CIP)	≥4	0	0	0
Gentamicin (GEN)	≥16	13 (18.3%)	0	1 (20%)
Kanamycin (KAN)	≥64	26 (36.6%)	0	1 (20%)
Nalidixic Acid (NAL)	≥32	0	0	0
Streptomycin (STR)	≥64	21 (29.5%)	0	1 (20%)
Sulfisoxazole (FIS)	≥256	37 (52.1%)	0	1 (20%)
Tetracycline (TET)	≥16	34 (47.8%)	0	1 (20%)
Trimethroprim/Sulfamethoxazole (SXT)	≥4/76	11 (15.4%)	0	0

**Table 3:** Resistance patterns among 51 *S*. Senftenberg recovered from animal and miscellaneous sources

Pattern	# of isolates with pattern
CHL	1
FIS	2
KAN	1
SXT	5
TET	1
FIS, TET	3
GEN, FIS	1
STR, SXT	3
STR, TET	1
STR, TET, SXT	4
TIO, TET	1
TIO, FIS, TET	1
KAN, FIS	1
KAN, STR, FIS	1
KAN, FIS, SXT	1
KAN, FIS, TET	3
KAN, STR, TET, SXT	1
KAN, FIS, TET, SXT	3
GEN, KAN, STR, FIS	1
GEN, KAN, STR, FIS, TET	1
GEN, KAN, STR, FIS, TET, SXT	1
AMP, KAN, STR, TET	1
AMP, KAN, STR, FIS, TET	1
AMP, GEN, KAN, FIS, TET	1
AMP, GEN, KAN, STR, FIS, TET	1
AMP, CHL, GEN, KAN, STR, FIS, TET	1
AMP, GEN, KAN, STR, FIS, TET, SXT	1
AUG, GEN, KAN, STR, TET, SXT	1
AUG, AMP, FOX, TIO, STR, FIS, TET, SXT	1
AUG, AMP, FOX, TIO, CHL, STR, FIS, TET	2
AUG, AMP, FOX, TIO, KAN, STR, FIS, TET, SXT	1
AUG, AMP, FOX, TIO, CHL, KAN, STR, FIS, TET, SXT	1
AUG, AMP, FOX, TIO, CHL, GEN, KAN, STR, FIS, TET, SXT	2

CHL - chloramphenicol, FIS - sulfisoxazole, KAN - kanamycin, SXT - trimethoprim/sulfamethoxazole, TET - tetracycline, GEN - gentamicin, STR - streptomycin, TIO - ceftiofur, AMP - ampicillin, AUG - amoxicillin/clavulanic acid, FOX - cefoxitin.

Simpson's Index of Diversity (*D*) was used to evaluate the results of PFGE, antimicrobial resistance profiling and sequence types from MLST analysis. The discrimination index was highest for antimicrobial resistance analysis (*D *= 0.472) followed by MLST (*D *= 0.25), and PFGE (*D *= 0.155).

The data demonstrates that there are at least two sequence types of *S. *Senftenberg circulating in both animal and human hosts. Of interest, our sequenced strain (3-70-11), identified as an ST 185, falls in the same cluster as isolates implicated in human disease and those recovered from animals. Also of interest, the majority of isolates identified as ST 14, which were found in both human and animal hosts, tested (diagnostic or healthy) were not exclusive to a single host. It was evident that the MLST sequence types did not provide as good a method of differentiation as that of PFGE when examined using Simpson's Index of Diversity (0.155 for PFGE versus 0.25 for MLST). The PFGE profiles, which were relatively unique among the strains tested, resulted in 93 profiles for the 98 strains tested. PFGE revealed some clustering but the majority of PFGE profiles appeared to be unique to the individual strains.

## Discussion

This study examined *S. *Senftenberg isolates from humans and animals to assess the genetic relatedness of *S. *Senftenberg from various hosts. In total, 98 strains of *S. *Senftenberg from various locations in the United States associated with humans and animal hosts were assessed using PFGE, MLST and antimicrobial susceptibility analysis (NARMS).

Pulsed field gel (PFGE) analysis of the isolates found that most *S. *Senftenberg isolates examined had profiles that appeared to be unique to the individual strains; among the 98 strains tested 93 unique profiles were identified. Cluster analysis identified four primary clusters at approximately 58% similarity; with most clusters composed of ST 14 and a single cluster consisting of ST 185. It was evident that PFGE provided greater differentiation than MLST alone which would have created two clusters only. This observation was supported by the diversity indices which found that PFGE resulted in the greatest rate of diversity over MLST and antimicrobial susceptibility testing. Similar studies by our lab investigating *S*. Typhimurium found that PFGE provided greater differentiation for the strains than MLST alone [5]. It has been suggested that housekeeping genes can be too conservative and greater differentiation may be possible by expansion of the panel to include virulence genes where inherent variation may be greater [6]. In a recent study, Liu et al [24] used two virulence genes (*sseL *and *fimH*) and a clustered regularly interspaced short palindromic repeat loci (CRISPR) as an alternative MLST analysis for subtyping the major serovars of *Salmonella enterica *sub species *enterica*. The MLST scheme using only the two virulence genes corresponded well with the serotypes but failed to discriminate between outbreak strains. Incorporation of the CRISPR sequences enhanced the discriminatory power to differentiate at the strain outbreak level, suggesting that modification of the MLST can enhance differentiation ability.

Antimicrobial susceptibility analysis of all isolates found that the human strains were susceptible to all of the antimicrobials of the NARMS panel; in contrast, the animal isolates showed a range of resistances with most isolates being resistant to two or more antimicrobials. The rate of resistance to antimicrobials was somewhat similar across the host species (13 porcine, 11 bovine and 12 poultry) with 11 isolates displaying resistance to 6 or more agents. Further studies to determine the nature of the resistance observed is ongoing but it is possible that mobile genetic elements such as integrons may be responsible for some of the high resistance levels observed in porcine isolates [38].

Sequence analysis of the isolates found that the most common sequence type (ST) observed among all isolates were ST 14 and ST 185, one isolate identified as ST 145 was recovered from a pig. ST 14 isolates were the most common being found in *S. *Senftenberg of porcine, equine, bovine, turkey, feline, canine, and human origin. Comparison of our data with the MLST database indicates that ST 14 is relatively common in a range of hosts including poultry, soya, fishmeal, lizard, and humans (http://www.mlst.net). Of interest, this ST has been found worldwide and is included in the SARB collection [39]. In contrast, the ST 185 isolates of this study were relatively unique and found only in a small collection of turkey, bovine, and human hosts. When compared with the MLST database, this strain type was not as common being found only in isolates associated with animal feed and humans and primarily among strains recovered in Europe.

While relatively little is known about *S. *Senftenberg, the organism does appear to be associated with human disease and has been found to persist in feed, and feed materials in feed factories as well as poultry, poultry farms and the processing environment [8,40-44]. Among CDC data, *S*. Senftenberg appears to be primarily associated with non-human clinical disease however, the organism has been associated with human illness and with a range of foods including fennel seed tea, nuts, herbs, baby cereal, poultry, and cattle and most recently spices [8,45,48-50,52] and appears to be emerging in plant and plant products [46,47,51]. One of the limitations of this study is that traits and characteristics of *S*. Senftenberg have only been assessed in animal and human isolates and it is unknown if these observations hold true for isolates of plants (herbs, spices etc.). It is also interesting to speculate as to the nature of *S*. Senftenberg as it appears to be an emerging strain in human illness and animals as both a commensal but possibly also as an opportunistic pathogen. Ongoing analyses in our lab may clarify further the nature and pathogenesis of this serotype.

## Conclusions

This study has highlighted the use of molecular and phenotype analysis for characterization of *S*. Senftenberg. Greatest diversity was observed among isolates using PFGE supporting its use as a subtyping method to differentiate isolates of the same serovar. Three sequence types were observed with MLST analysis and types were not host specific. Antimicrobial resistance was evident in animal isolates but not human reflecting the nature of animal husbandry.

## Abbreviations

AMI: Amikacin; AUG: amoxicillin/clavulanic acid; AMP: ampicillin; AXO: ceftriaxone; CHL: chloramphenicol; CIP: ciprofloxacin; FOX: cefoxitin; FIS: sulfisoxazole; GEN: gentamicin; KAN: kanamycin; MLST: multilocus sequence type analysis; NAL: nalidixic acid; NARMS: national antimicrobial resistance monitoring system; PCR: polymerase chain reaction; PFGE: pulsed field gel electrophoresis; STR: streptomycin; SXT: trimethoprim/sulfamethoxazole; TET: tetracycline; TIO: ceftiofur.

## Competing interests

The authors declare that they have no competing interests.

## Authors' contributions

RMS - carried out the MLST and NARMS analysis of all isolates used in the study; JSS carried out NARMS and PFGE analysis; SRP was involved in isolate screening; CML devised the project, wrote and edited the paper with input from RMS, JSS and SRP. All authors read and approved the final manuscript.
